# Analysis and improvement of the three-column spinal theory

**DOI:** 10.1186/s12891-020-03550-5

**Published:** 2020-08-12

**Authors:** Qihang Su, Cong Li, Yongchao Li, Zifei Zhou, Shuiqiang Zhang, Song Guo, Xiaofei Feng, Meijun Yan, Yan Zhang, Jinbiao Zhang, Jie Pan, Biao Cheng, Jun Tan

**Affiliations:** 1 Department of Orthopedics, Shanghai East Hospital, Tongji University School of Medicine, China. No.150 Jimo Road, Shanghai, 200120 China; 2 Department of Orthopedics, Shanghai Tenth People’s Hospital, Tongji University School of Medicine, Shanghai, 200072 China; 3 Department of Trauma Surgery, Shanghai East Hospital, Tongji University School of Medicine, China. No.150 Jimo Road, Shanghai, 200120 China; 4 School of Engineering, Huzhou University, Huzhou, 313000 China; 5 Department of Orthopedics, Pinghu Second People’s Hospital, Pinghu, 314200 China

**Keywords:** Vertebral fractures, Three-dimensional mapping, Finite element force analysis, Three-column spinal theory

## Abstract

**Background:**

Denis and Ferguson et al.’s three-column spinal theory has been widely accepted and applied. However, this three-column theory was proposed based solely on observation and experience without thorough documented data and analysis. The aim of this study was to analyze and improve Denis and Ferguson et al.’s three-column spinal theory to propose a novel three-column concept in epidemiology, morphology and biomechanics.

**Methods:**

A retrospective analysis of the computed tomography imaging data of patients with a diagnosis of T11-L5 vertebral fractures was conducted between February 2010 and December 2018. Three-dimensional (3D) distribution maps of fracture lines of all subjects were obtained based on 3D mapping techniques. In addition, a 25-year-old health male volunteer was recruited for the vertebral finite element force analysis.

**Results:**

The present study enrolled 459 patients (age: 48 ± 11.42 years), containing a total of 521 fractured vertebrae. The fracture lines peaked in the upper and the outer third sections of the vertebra, starting from the anterior part of the vertebral pedicles in 3-D maps. Regarding flexion and extension of the spine, the last third of the vertebral body in front of the spinal canal was one main stress center in the finite element analysis. The stress on the vertebral body was greater in front of the pedicles in the lateral bending.

**Conclusion:**

The study reveals that the posterior one-third of the vertebral body in front of the spinal canal and the posterior one-third of the vertebral body in front of the pedicle are very different in terms of fracture characteristics and risks to spinal canal (3D maps and stress distributing graphs), therefore, they should be classified as different columns. We provide strong evidence that Su’s three-column theory complies with the characteristics of vertebral physiological structure, vertebral fracture, and vertebral biomechanics.

**Graphical abstract:**

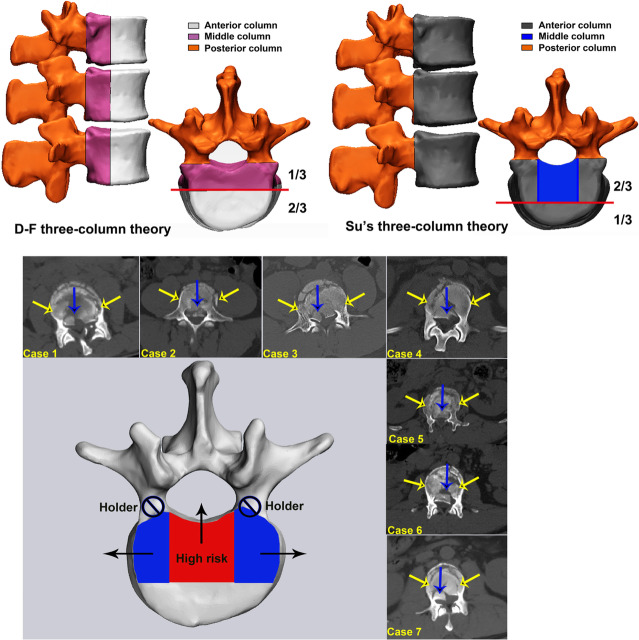

## Background

Thoracolumbar (TL) spine fracture is the most common type of osteoporotic fractures, followed by lumbar and cervical spine fractures, which account for almost 90% of all spinal injuries [1]. Many classification systems of TL spinal fractures have been proposed to facilitate communication among clinicians and enhance treatment protocols, but none have achieved universal adoption. Holdsworth’s classification system of TL injuries has had a major impact on the mechanistic studies and introduced the concept of the “vertebral column” [2, 3]. Kelly and Whitesides have developed a two-column concept of spine stability and used the analogy of a construction crane to illustrate this mechanical principle, in which the boom being the pressure-resistant vertebral bodies and discs (anterior column), and the guy rope being the posterior vertebral elements and ligaments resisting tension (posterior column) [4, 5].

**Fig. 1:**
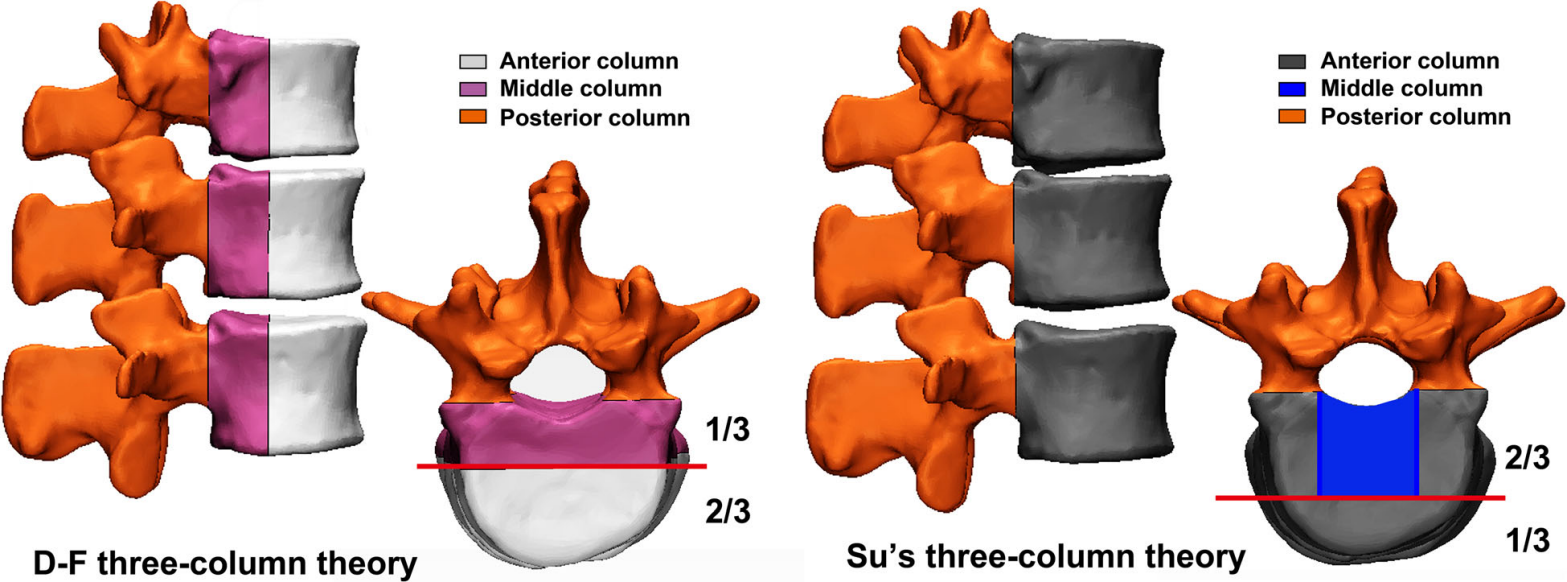
The pattern diagram of three-column spinal theory. D-F three-column theory was initially established by Denis and Ferguson et al. Su’s three-column theory was a novel three-column concept proposed by our team

A three-column concept has been initially introduced to establish a morphological classification system comprising of vertebral bodies and 2 rows of intra-articular masses [6]. However, this three-column concept is not universally accepted. Unlike the above-mentioned concepts, the posterolateral part of the anterior column has been deemed to be a key structure of spinal instability, especially during flexion [7–9]. Consequently, the initial anterior column was separated to 2 sections by introducing a middle column, which was then modified by Ferguson and Allen [10]. Following this development, the D-F three-column theory (Denis and Ferguson et al.) [7–10] was widely accepted and applied, which composed of the anterior 2/3 and posterior 1/3 of the vertebral body, the anterior 2/3 and posterior 1/3 of the annulus fibrosus, the anterior and posterior longitudinal ligaments, the posterior bony complex (posterior arch) alternating with the posterior ligamentous complex (capsule, interspinous ligament, ligamentum flavum and supraspinous ligament) (Fig. 1).

This three-column theory was proposed based solely on observation and experience without thorough supporting data. Therefore, using the recurrent patterns of T11-L5 vertebral fracture characteristics in terms of epidemiology, distribution maps of fracture lines based on three-dimensional (3-D) computed tomography (CT) mapping techniques (Morphology), and vertebral finite element (FE) force analysis (Biomechanics), this study aimed to improve the current D-F three-column theory by developing a novel three-column concept (i.e., Su’s three-column theory). The vertebral bodies were subdivided equally into three parts, and two parallel extension lines were then drawn along the medial edges of the pedicles to the first-third of the vertebral bodies, as shown by the blue region in Fig. 1. The anterior longitudinal ligament was used to generate the anterior column, in addition to a portion of the vertebral bodies and the fibrous ring in the dark grey region. The middle column was composed of the posterior longitudinal ligament combined with a portion of the vertebral bodies and fibrous ring in the blue region. The posterior column remained essentially the same as described by Denis [7–9]. Consequently, we hypothesized that Su’s three-column theory is more consistent with the characteristics of vertebral anatomy, vertebral fracture patterns, and vertebral biomechanics.

## Methods

### Patient cohort

The CT imaging data of patients diagnosed with T11-L5 vertebral fractures were retrospectively analyzed from February 2010 to December 2018. The fracture vertebral body of T11-L5 was the region of interest. Inclusion criteria were as follows: (i) age: 18–65 years old; (ii) fracture classification: type-A fractures of T11-L5 vertebral body, except for Type A1.1 and A1.3, according to Magerl’s AO system [11]; (iii) non-osteoporotic and non-pathologic vertebral fractures; and (iv) high quality of CT imaging available. Patients were excluded if they had: (i) fracture lines or anatomical landmarks obscured by foreign bodies; and (ii) severely comminuted fractures or depressed/impacted fractures, where the fracture lines are difficult to be determined. All subjects were evaluated by three experienced spinal clinicians (QHS, CL and YCL) and the corresponding author (JT), utilizing the initial 3-D CT stored in the Picture Archiving and Communication System database.

### Fracture mapping

**Fig. 2:**
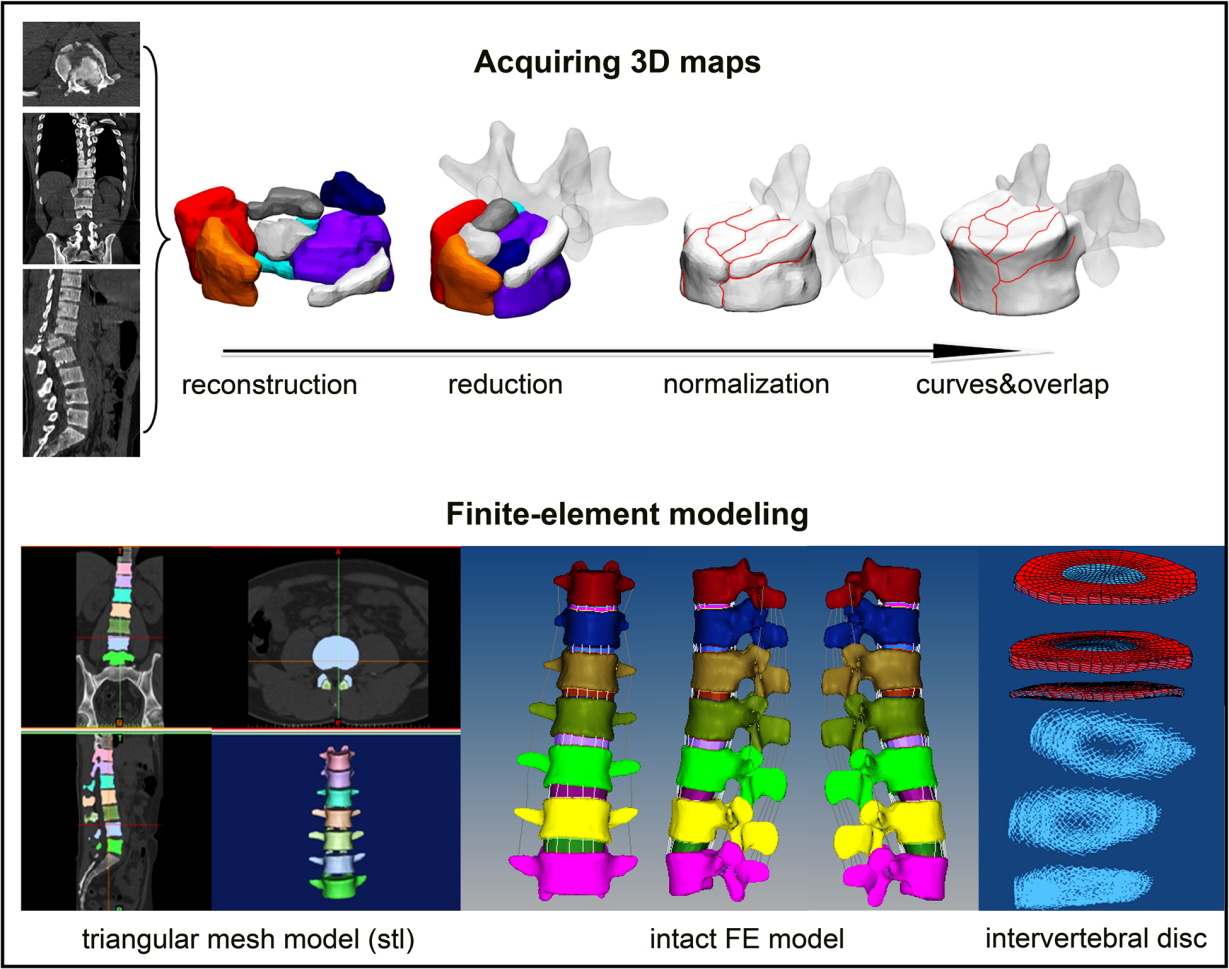
The procedures of 3D fracture mapping and finite-element modeling

As mentioned above, the fracture of T11-L5 vertebral body was our region of interest. The 3-D fracture maps of T11-L5 vertebral body (Fig. 2) were constructed as described previously [12, 13]. Raw data in the DICOM (Digital Imaging and Communications in Medicine) file format were obtained using a Siemens SOMATOM Sensation 64-channel CT scanner (Siemens, Erlangen, Germany). The parameters used were as follows: tube current = 200 mA, tube voltage = 120 kV, interlayer spacing = 0.5 mm, and slice thickness = 1 mm. Subsequently, the DICOM data were imported into Mimics version 20.0 software (Materialise Inc., Leuven, Belgium) to reconstruct and virtually reduce the 3-D fracture fragments by adjusting the obtained tissue threshold. Next, the reconstructed fragments were exported into 3-matic version 9.0 software (Materialise Inc., Leuven, Belgium) to optimally match the 3-D model of the template vertebral body via reduction of fragments. After that, curves were directly drawn onto the model’s surface for delineating the fracture lines of each vertebrae. Lastly, all the fracture lines were overlapped to generate a 3-D fracture map of T11-L5 vertebral body.

### Finite element analysis

A 25-year-old health male volunteer (height, 173 cm; weight, 67 kg; body mass index, 22.386 kg/m^2^) was recruited for a computed tomography scan from T11 to L5 levels using a 64-channel CT scanner. This participant provided written informed consent to participate in this study.

DICOM data from CT scanning of all subjects were imported into Mimics software, and the 3D reconstruction models of T11-L5 vertebral were obtained by threshold segmentation using the STL format. The generated STL data were then imported into Geomagic Studio (V2014, Geomagic, USA) and HyperMesh (V14.0, Altair, USA) to adjust and optimize the model. Subsequently, the Initial Graphic Exchange Specification (IGES) format entity model was generated. The generated virtual model was imported into Hypermesh, a pre-processing software for finite element mesh generation, and then subdivided into tetrahedral meshes with a thickness of 1 mm (C3D4). The outermost mesh of the vertebral body was separated from the interior as the cortical bone of the vertebral body, and the rest was denoted as cancellous bone. Based on the upper and lower sides of adjacent vertebral bodies, the hexahedral intervertebral disc mesh was established. The upper and lower layers of mesh were used as the endplate of the intervertebral disc. Among them, the outer eight layers were used as the fibrous ring, and the rest were the nucleus pulposus. The complete intervertebral disc including the endplate, the fibrous ring and the nucleus pulposus, was reconstructed accordingly. The meshed vertebral bodies were connected by T3D2 units, isotropic linear elastic material with tension only without compression, to simulate the ligament effect. The fibrous ring is composed of fibrous ring matrix and collagen fiber. The matrix of fibrous ring was developed with a solid unit, and the collagen fiber uses a truss unit. The collagen fibers of the fibrous ring were only subjected to tensile stress but not pressure; the fibers crossed each other at an angle of about 30° and an average angle of ±29° with the intervertebral disc, and were arranged in the form of eight concentric layers, accounting for 16% of the fibrous rings [14] (Fig. 2). The materials for the various parts of the T11-L5 model and their characteristics, including elastic modulus and Poisson ratio, are listed in Table 1.

**Table 1:** Material property of spinal components

Material	Young’s Modulus (MPa)	Poisson’s Ratio	Area (mm^**2**^)	Element type	Element numbers
Cortical bone	12,000.00	0.30	–	C3D4	340,465
Cancellous bone	100.00	0.20	–	C3D4	413,282
Endplate	25.00	0.25	–	C3D8R	46,849
Nucleus pulposus	0.20	0.49	–	C3D8R	84,761
Annulus ground substance	4.00	0.40	–	C3D8R	163,585
Annulus fiber	4.20	0.45	–	truss	726
Anterior longitudinal ligaments	20.00	0.30	63.70	spring	30
Posterior longitudinal ligaments	20.00	0.30	20.00	spring	30
Intertransverse ligament	58.70	0.30	3.60	spring	30
Ligamentum flavum	19.50	0.30	40.00	spring	30
Interspinous ligament	11.60	0.30	40.00	spring	12
Supraspinous ligament	15.00	0.30	30.00	spring	3
Capsular ligament	32.90	0.30	60.00	spring	30

In the three-dimensional finite element model of the T11-L5 vertebral body generated in the present study, the joint between vertebral body and intervertebral disc was defined as Tie. The interaction of joint surfaces between the upper and lower articular processes of vertebral body was considered as contact, and the friction coefficient was defined as 0.1. The boundary conditions during model calculation include a fixation support on the inferior surface of L5 and a bending moment applied on the upper surface of T1. In the actual process of spinal traction, the waist of the patient is fixed, so the fixation support of L5 inferior surface is set in the model to limit its translation and rotation, so that it remains stable during loading. In the model, the coupling constraint is applied on the upper surface of T11, and the bending moment is applied on the reference node of the coupling constraint; the bending moment is 7.5 Nm and the downward axial compression force is 500 N to simulate the stress when the spine is moving in different directions under self-weight stress [15].

Through static analysis of the FE model via the finite element method, the stresses of the FE model under forward flexion, backward extension, left and right lateral bending, left and right torsion were obtained. Accordingly, von Mises analysis (stress distributing graphs) was applied as the stress observation index in the study. This intact T11-L5 FE model was validated by comparing the intact finite-element model reported in previous studies [16–18] and is shown in Fig. 2. The boundary conditions and loads to the finite element model were applied to simulate the left bending, right bending, flexion, extension, left rotation and right rotation of lumbar vertebrae. The model for healthy lumbar vertebra was calculated to acquire the range of motion (ROM). The mobility data were compared with the in-vitro biomechanical experimental data in previous literatures, and the accuracy and effectiveness of the model were verified by the consistency of mobility [19–21].

## Results

**Fig. 3:**
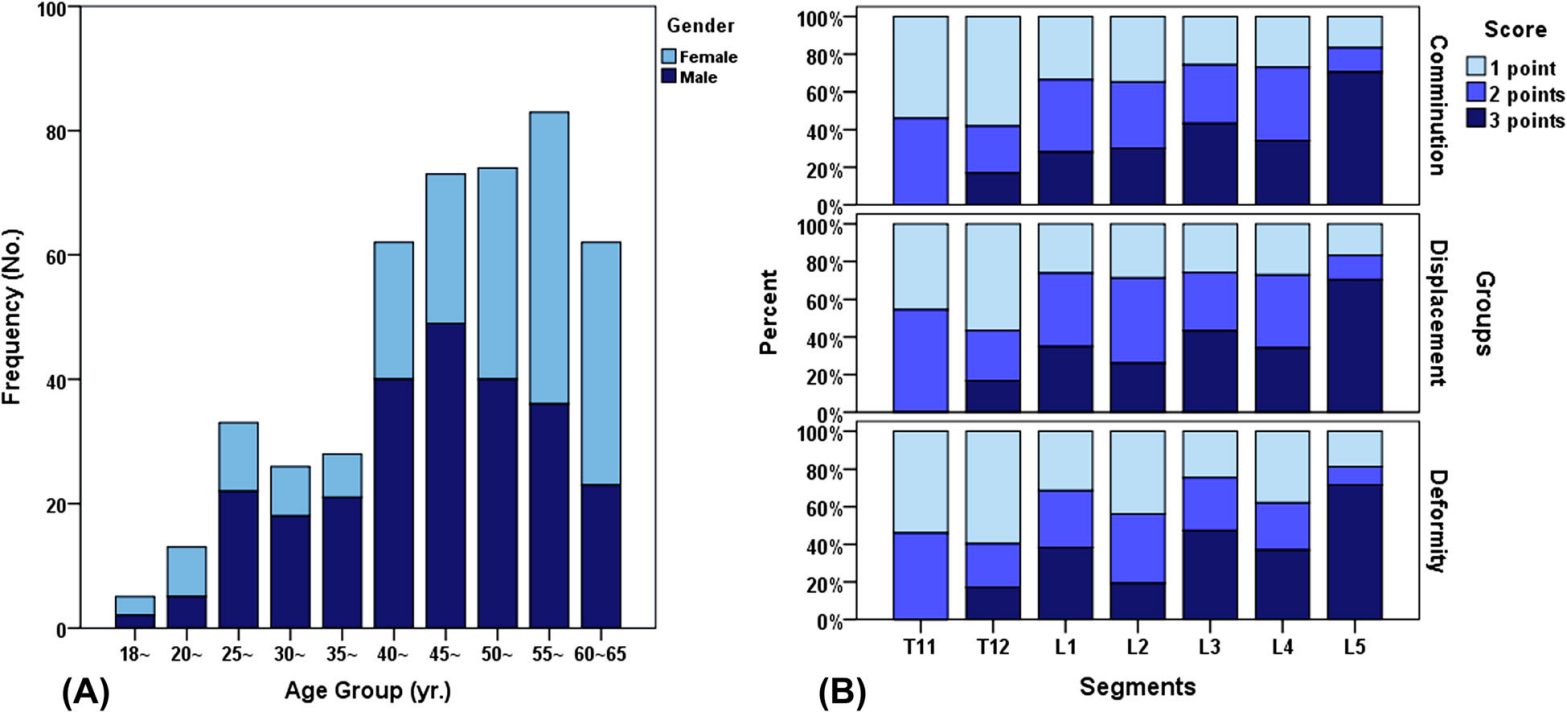
**a** The histogram of age groups of all cases. **b** The percentage distribution diagram for the McCormack et al.’s LSC scoring of 521 fracture vertebrae: the amount of comminution/involvement, the amount of apposition/displacement of fracture fragments, and the amount of correction of kyphotic deformity

The present study enrolled 459 patients, containing a total of 521 cases of vertebral fracture, and a healthy male volunteer. There were 256 male and 203 female patients, with a mean age of 48 years (SD = 11.42). Patient demographics are summarized in Table 2 and age distribution is shown in Fig. 3a.

**Table 2:** Patient Demographics (*N* = 459)

Variables	
Gender (no.)	
Male (%)	256 (55.77)
Female (%)	203 (44.23)
Mean age ± SD (yr)	48 ± 11.42
Mechanism of Injury (no.)	
Fall (%)	315 (68.63)
Traffic accident (%)	144 (31.37)
Total fracture vertebrae (no.)	521
T11	13
T12	73
L1	171
L2	143
L3	64
L4	38
L5	19

AO fracture classifications are shown in Table 3, from which Type A3.1.1 and A1.2.1 accounted for the vast majority. In addition, we also used McCormack et al.’s load sharing classification (LSC) (Fig. 3b) to assess the characteristics of these vertebral fracture cases [22]. It can be inferred that T11 and T12 vertebral bodies were more likely to have less severe damage under most circumstances, i.e. comminution/involvement, displacement/apposition of fracture fragments, and correction of kyphotic deformity, which were predominantly scored as one. Other vertebrae, L5 vertebrae in particular, were scored as 2 or 3 points in most cases.

**Table 3:** Fracture Distribution (*N* = 521)

Groups (Type A.)	T11	T12	L1	L2	L3	L4	L5	Total
A1. Impaction fractures	8	42	91	82	29	18	8	278
A1.2.1	8	37	83	77	28	17	8	
A1.2.2	0	3	4	2	0	0	0	
A1.2.3	0	2	4	3	1	1	0	
A2. Split fractures	0	2	7	4	2	2	1	18
A2.1	0	1	4	0	1	1	1	
A2.2	0	1	2	3	1	1	0	
A2.3	0	0	1	1	0	0	0	
A3. Burst fractures	5	29	73	57	33	18	10	225
A3.1.1	3	14	39	36	16	12	3	
A3.1.2	0	3	7	7	5	1	1	
A3.1.3	1	1	5	2	1	1	0	
A3.2.1	1	6	9	5	3	2	0	
A3.2.2	0	1	2	1	0	0	0	
A3.2.3	0	0	1	0	1	0	0	
A3.3.1	0	0	4	0	2	1	2	
A3.3.2	0	2	4	2	3	0	3	
A3.3.3	0	2	2	4	2	1	1	

### 3D maps

**Fig. 4:**
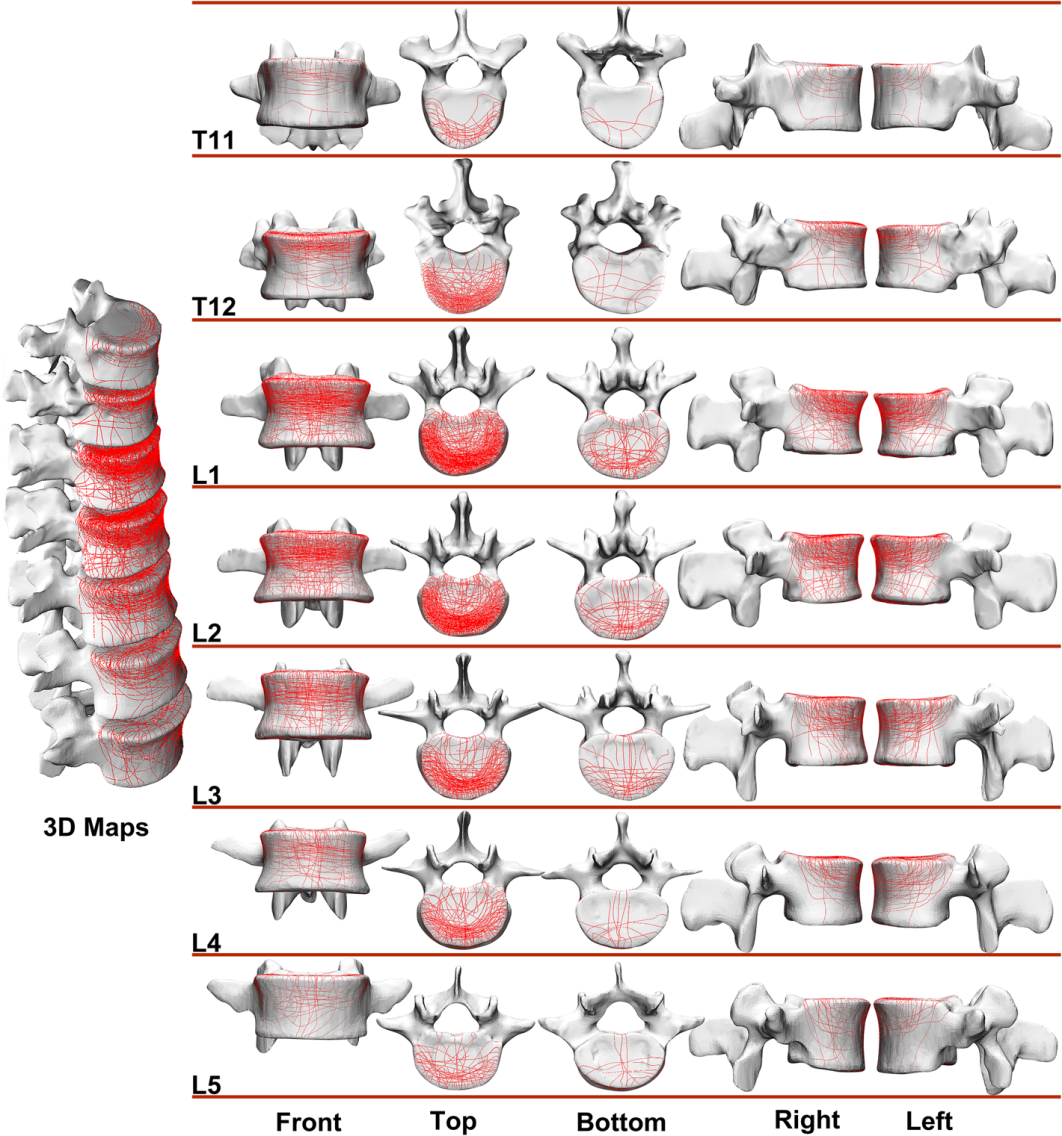
The 3D maps of T11-L5: the front view, the top view, the bottom view, the right view and the left view (from left to right)

The 3D maps of T11-L5 display the fracture line distribution of the vertebral body (Fig. 4). The fracture lines of these 3-D maps peaked in the upper and the outer third sections of the vertebra, starting from the anterior part of the vertebral pedicles, and were also arranged in annular wedges along the front and side of the vertebral body.

### Finite element analysis

**Fig. 5:**
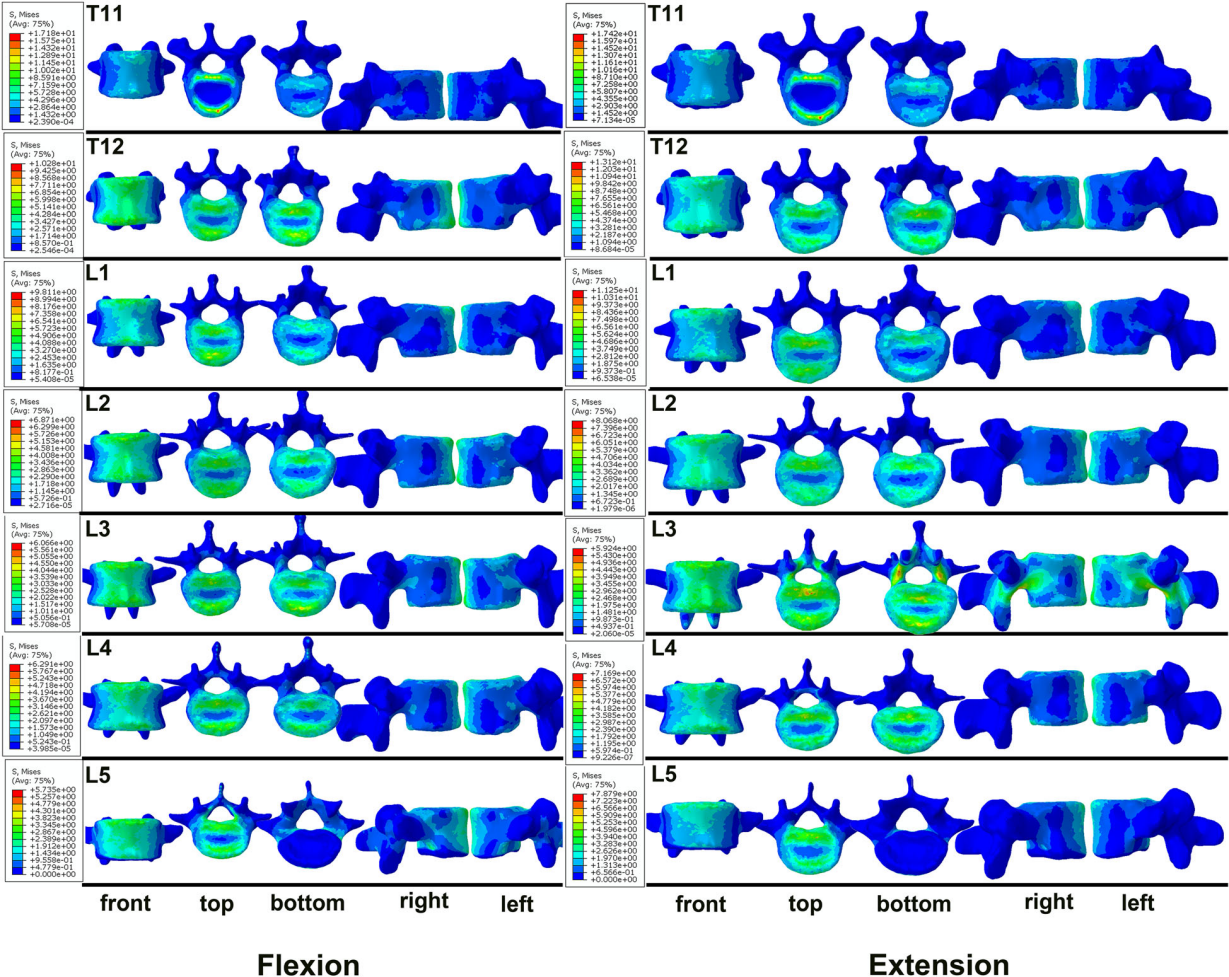
The stress distributing graphs of T11-L5 under forward flexion and backward extension: the front view, the top view, the bottom view, the right view and the left view (from left to right)

**Fig. 6:**
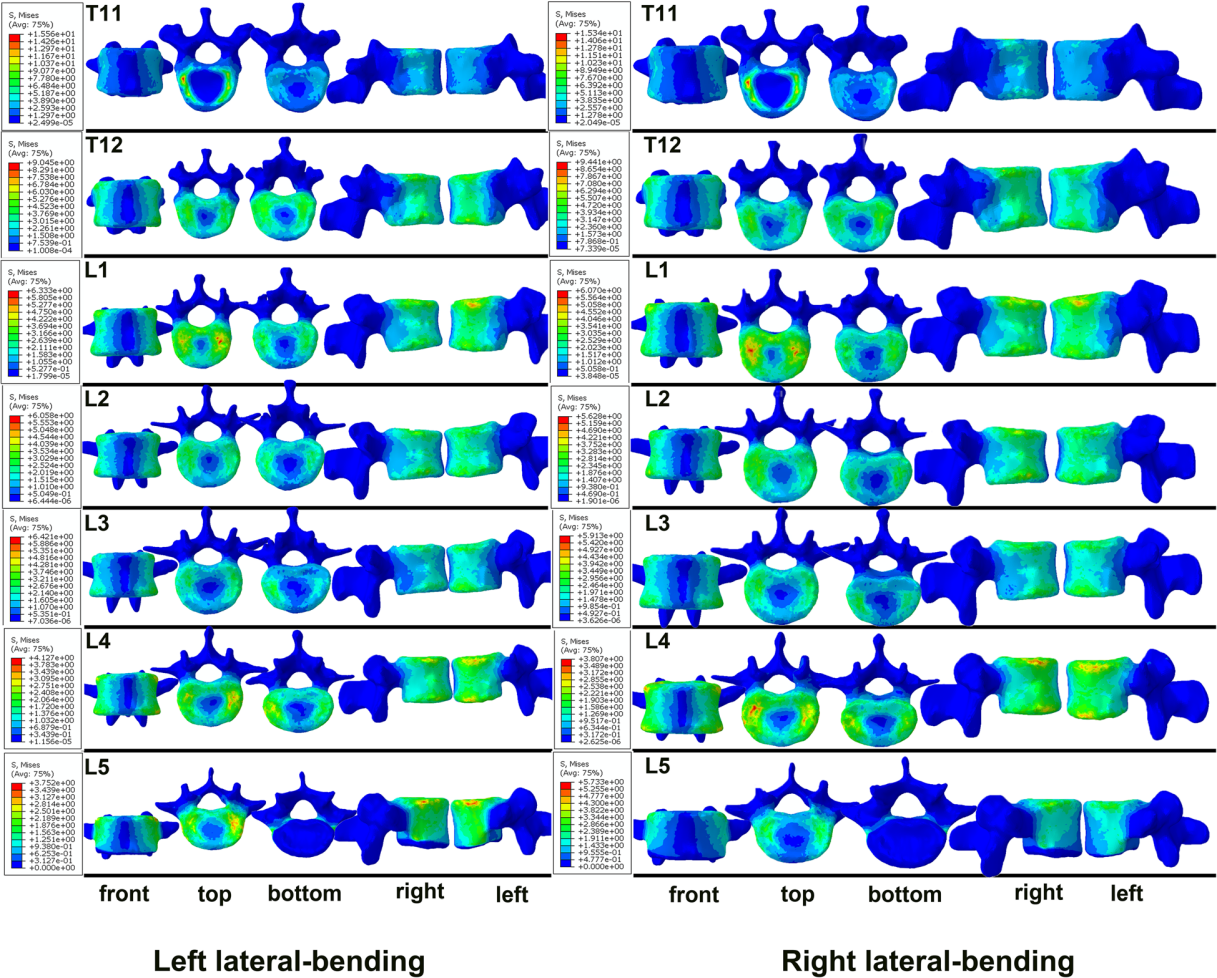
The stress distributing graphs of T11-L5 under left and right lateral bending: the front view, the top view, the bottom view, the right view and the left view (from left to right)

**Fig. 7:**
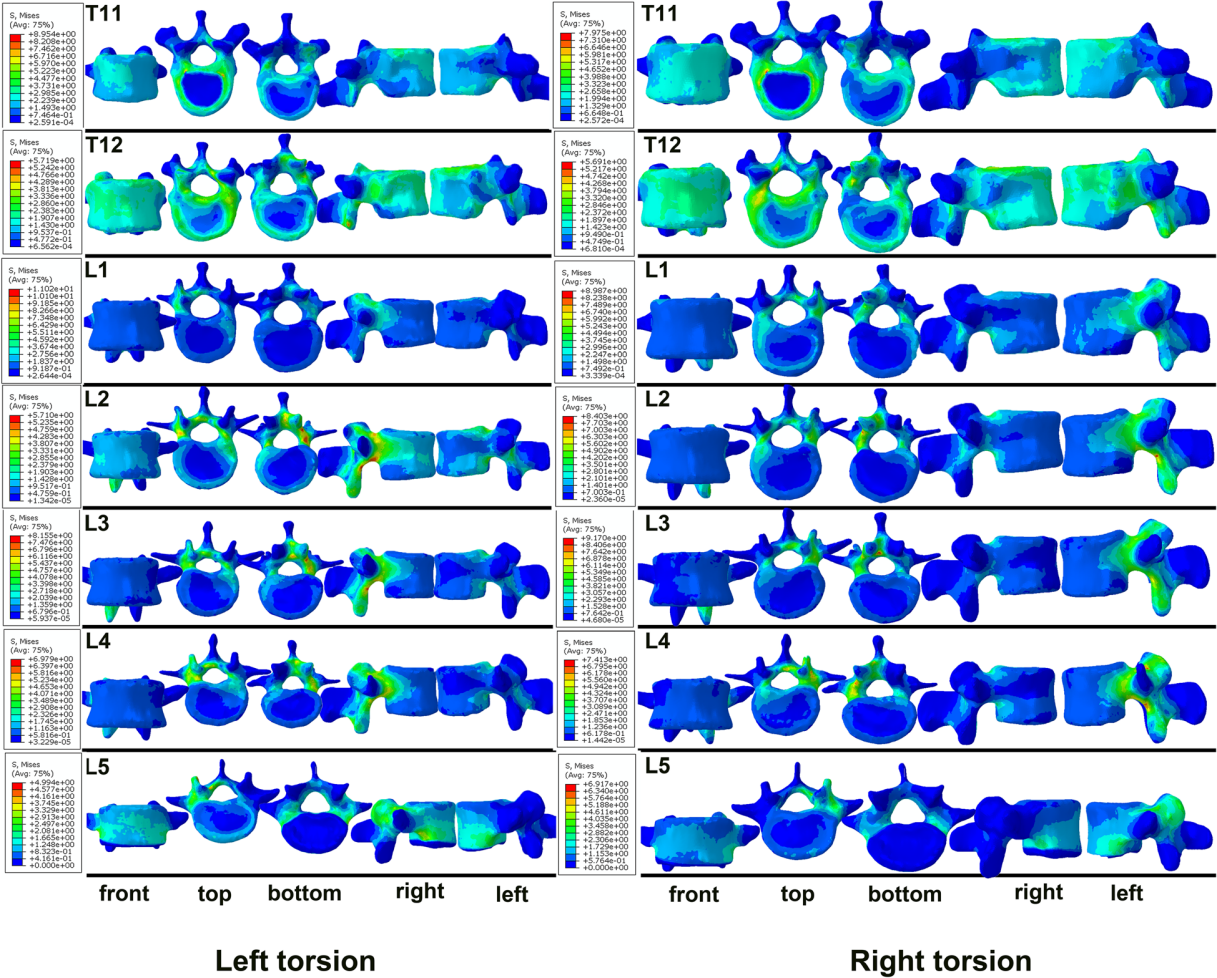
The stress distributing graphs of T11-L5 under left and right torsion: the front view, the top view, the bottom view, the right view and the left view (from left to right

Figure 5, 6 and 7 displays the forces acting on each vertebral body under flexion, extension, lateral bending and torsion. Regarding flexion and extension of the spine, there were two main stress centers in the vertebral body, namely the first third and the last third of the vertebral body in front of the spinal canal. In contrast, the middle of the vertebral body and the vertebral body in front of the pedicles were under less stress (Fig. 5). In terms of lateral bending, the stress on the vertebral body was greater in front of the pedicles on both sides (Fig. 6). For spine torsion, the stressed area was mainly concentrated on the posterior column of the vertebral body, while the stressed area of the vertebral body was smaller, and the stressed area was mainly concentrated on the edge of the vertebral body in a circular pattern (Fig. 7).

## Discussion

The three columns characterized in the present study do not anatomically or biomechanically resemble a real column [10]. They represent an abstract concept proposed to characterize spinal injuries, serve as a classification system, and emphasize the value of these three regions in the maintenance of spinal stability. In addition to observation and experience, thoroughly documented data including epidemiology, morphology and biomechanics are needed to define and optimize the three-column theory to enhance clinical decision-making and scientific research.

**Fig. 8:**
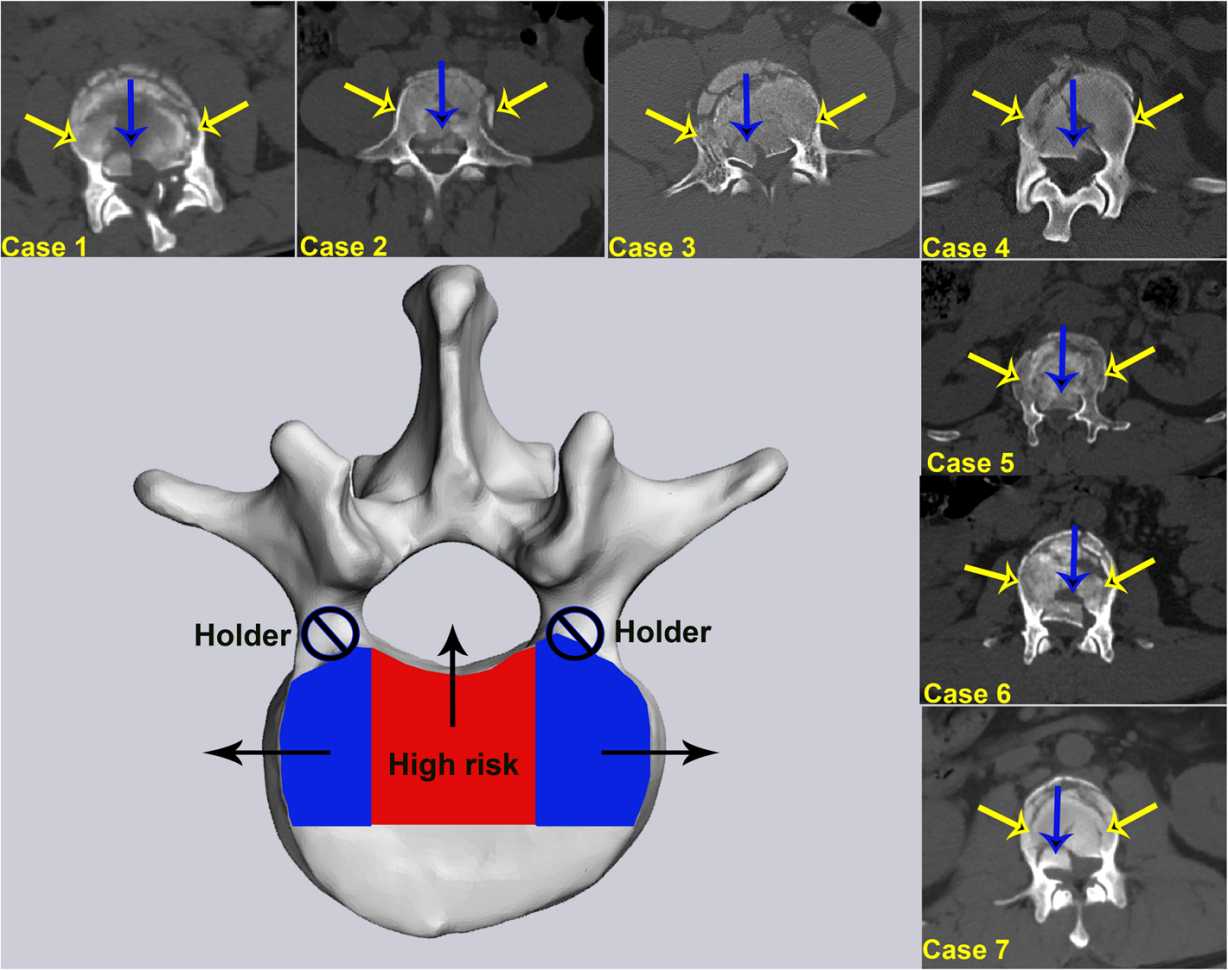
Compared with the posterior part of the vertebral body in front of the pedicle (blue region and yellow arrows), the posterior part of the vertebral body in front of the spinal canal (red region and blue arrows) posed the higher risks spinal canal

From an epidemiological perspective, Type A.1.2 wedge impaction fractures were the most common subtype. Magerl et al. proposed that wedge deformation occurs in this subtype of fracture, but the posterior wall of the vertebral body remains intact [11]. The posterior wall of the vertebral body is typically considered as two parts, i.e., the posterior wall of the vertebral body in front of the spinal canal and the posterior wall of the vertebral body in front of the pedicle. However, the current results indicate that the posterior wall of the vertebral body in front of the spinal canal remains intact in many cases of wedge fracture, but many fractures involve the posterior wall of the vertebral body at the front of the pedicle, especially lateral wedge fractures. The second most common type of fracture is the superior incomplete burst fracture, where the greatest risk is that fragments of the posterior wall of the vertebral body may be further retropulsed into the spinal canal when the injury is exposed to flexion/compression [4]. Similarly, we still need to assess the effects on the posterior part of the vertebral body, especially considering questions about whether the posterior part of the vertebral body in front of the spinal canal (RED) and the posterior part of the vertebral body in front of the pedicle (BLUE) pose the same risks to spinal canal, as a whole (Fig. 8).

Based on big-data analyses (3D Maps) of morphological features, it is clear that the fracture line of T11-L5 vertebra is circularly distributed along outer third of the vertebral body, and many fracture lines extend to the posterior part of the vertebral body in the front of the pedicle, that is, the anterior part of the pedicle in the middle column based on the D-F three-column theory. Although there are fewer cases of T11 and L5 fracture, patterns in these injuries can be discussed generally.

Through morphological analysis, clinical experience and literature review [23–27], we observed a low incidence of fracture of vertebral body in front of the pedicle where other regions remain intact. In many cases, fractures occur in the first third of the vertebral body, which affects the BLUE. However, the incidence of fractures along the RED that other regions remain intact is relatively high, and most fractures with severe complications caused by the protrusion into the spinal canal are located in this subregion. In contrast, vertebral fractures in front of the pedicle rarely cause severe complications due to the pedicle hold (Fig. 8). This phenomenon can be more fully explained through biomechanical analyses.

This intact T11-L5 FE model is valid because the ROM predicted by the present FE model matched well with the results of in vitro and other FE studies [15, 19–21]. The stress distribution graph for T11-L5 indicates that the posterior part of the anterior vertebral body of the spinal canal is the main stressed area under conditions of flexion and extension, and that part of the stress is transmitted to the pedicle during of L3 extension. Therefore, compared with the RED, the stress on the BLUE, as well as the threat to the spinal canal, is relatively minor, which may be attributed to the role of the pedicle holding and distributing the stress. Furthermore, in terms of the lateral bending state, the stress difference between the BLUE and the RED is more apparent, where the former is much larger than the latter. Given the reasons mentioned above, we suggest that the D-F three-column theory, which classifies the posterior one-third of the vertebral body in front of the pedicle and the posterior one-third of the vertebral body in front of the spinal canal as the mid-column, has limitations.

Therefore, we propose a modified (Su’s) three-column theory based on big-data analyses. There are substantial data to support the hypothesis that our modified three-column theory is more consistent with the physiological structure, fracture characteristics and biomechanical features of the spine. Based on epidemiological analysis and clinical experience, fractures without internal surgical fixation in the anterior column area are more likely in terms of Su’s three-column theory. Among these, fractures in the middle column often require surgical treatment to provide stability, and fracture fragments pose a risk to the spinal canal and cause serious complications. In addition, most fracture lines are concentrated in the front column based on the Su’s three-column theory; however, the results of stress analyses indicated that the force exerted by the posterior sagittal movement of the vertebral body in front of the pedicle was not great. Thus, it can be inferred that fractures of this particular part of the vertebral body are often caused indirectly by the fracture of the anterior one-third of the vertebral body, rather than directly by the stress upon this part of the vertebral body. It is therefore reasonable to classify these areas together as the anterior column. When the spine moves along the sagittal plane, the posterior part of the vertebral body in front of the spinal canal has its own stress center. In contrast, when moving along the coronal plane, the stress center shifts to the vertebral body in front of the pedicle instead. Overall, the distinctive posterior parts of the vertebral body are different from each other in terms of fracture characteristics and risks to spinal canal; therefore, the RED and the BLUE should be classified as different columns.

The D-F three-column theory and Su’s three-column theory have certain common characteristics: (1) posterior columns that are both defined precisely by the same concept; (2) the substantially greater mid-column contribution to the stability of the spine and risks to the spinal canal compared to the other two columns, which is often a critical factor affecting clinical decision-making. However, these two theories have differing definitions of the anterior and middle columns. We provide strong evidence that Su’s three-column theory complies with the characteristics of vertebral physiological structure, vertebral fracture, and vertebral biomechanics. We postulated that its use can result in enhanced guidelines for clinical decision-making and better characterization of spinal injuries, as well as provide an improved classification system of vertebral fractures. Too few spine models and the lack of different loading conditions in the finite element analysis are also limitations of the study. Therefore, the continuation of studies on the novel three-column concept in the finite element analysis will further reveal and improve the significance of the three-column concept.

Although Magerl et al.’s AO fracture classification system [11] is complex, it has many subtypes, which include all the fracture types, making the classification and summarization of vertebral fracture simpler. Therefore, this classification system was applied in this study for statistical analysis to understand and evaluate the epidemiological characteristics of vertebral fracture and fracture line distribution more comprehensively. However, the classification system developed by Magerl is limited in clinical application [28]. In 2013, Vaccaro et al. [29] published a new AO thoracolumbar spine injury classification system which reduces some fracture subtypes and combines thoracolumbar injury classification and severity score (TLICS) [30] to improve its clinical application value. The Type B and Type C in this system involve damage of the posterior column, and this study agrees with the D-F three-column theory and many other related studies [1, 7–9, 28–31] concerning the value of the posterior column, and so Type A was mainly discussed. In Vaccaro et al.’s new AO thoracolumbar spine injury classification system, Type A was divided into 4 types, with the fracture of the posterior vertebral wall involved in Type 3 and 4 but not in Type 1 and 2, which was consistent with the posterior wall of the D-F three-column theory. It is postulated that this classification mainly considers the threat of the posterior wall to spinal canal. Therefore, according to the previous analysis of this study, we believe that this classification is not highly appropriate. Due to the fixation effect of the pedicle, there is a significant difference in the posterior vertebral wall in front of the pedicle and that in front of the spinal canal contributing to high risks to the spinal canal and the stability of spine. For example, it is unclear if marginal fractures with the fracture line spreading from the anterior column to the posterior vertebral wall of the pedicle (the anterior column of the Su’s three-column theory) should be classified as Type 1 or 2 fractures.

As indicated by Vaccaro et al.’s new AO thoracolumbar spine injury classification system [29], clinical decision-making requires an assessment of a variety of factors, including fracture types, conditions of important nerves, conditions of muscles and ligaments, and even patients’ rapid rehabilitation needs, cultural differences, and medical levels. Therefore, this study cannot provide complete or accurate clinical decision-making recommendations. However, the analysis of this study reveals critical differences between the anterior column and the middle column, as well as the posterior vertebral wall in front of the pedicle and that in front of the spinal canal. Clinicians and researchers may be able to more comprehensively understand the importance of fractures involving the posterior vertebral wall in front of the pedicle (anterior column in Su’s three-column theory and middle column in the D-F three-column theory), and consider revising previous classification schemes and early internal fixation interventions.

## Conclusions

Denis and Ferguson et al.’s three-column theory, which classifies the posterior one-third of the vertebral body in front of the pedicle and the posterior one-third of the vertebral body in front of the spinal canal as the mid-column, has limitations. We provide strong evidence that Su’s three-column theory complies with the characteristics of vertebral physiological structure, vertebral fracture, and vertebral biomechanics.

## Data Availability

The raw data are available upon reasonable request from the corresponding author (Jun Tan).

## Abbreviations

3D: Three-dimensional
TL: Thoracolumbar
D-F: Denis and Ferguson et al.
CT: Computed tomograph
FE: Finite element
DICOM: Digital Imaging and Communications in Medicine
IGES: Initial graphic exchange specification
ROM: Range of motion
SD: Standard deviation
LSC: Load sharing classification
TLICS: Thoracolumbar injury classification and severity score
